# Cutaneous Manifestations in Patients With COVID-19 Treated at a University Hospital in Southern Brazil

**DOI:** 10.7759/cureus.31566

**Published:** 2022-11-16

**Authors:** Letícia Dupont, Rodrigo P Duquia, Gustavo W Pizutti, Fernanda B Nunes, Gisele Branchini, Elsa Stella B Mosquera, Renan R Bonamigo

**Affiliations:** 1 Postgraduate Program in Pathology, Universidade Federal de Ciências da Saúde de Porto Alegre, Porto Alegre, BRA; 2 Dermatology Service, Santa Casa de Misericórdia de Porto Alegre, Porto Alegre, BRA; 3 Dermatology, Universidade Federal de Ciências da Saúde de Porto Alegre, Porto Alegre, BRA; 4 Dermatology Service, Universidade Federal do Rio Grande do Sul, Porto Alegre, BRA

**Keywords:** covid-19 retro, sars-cov-2 infection, risk of covid 19 mortality, risk factors, cutaneous manifestations, covid-19

## Abstract

Objectives

The aim of this study was to ascertain whether pattern of cutaneous lesions, age, sex, ethnicity, long-term medication use, arterial oxygen saturation at the first examination, setting of care, and number of medications used to treat SARS-CoV-2 infection are associated with mortality in patients with a confirmed diagnosis of coronavirus disease 2019 (COVID-19) and cutaneous manifestations. In addition, to evaluate the occurrence of cutaneous manifestations in patients with a confirmed diagnosis of COVID-19 through a review of medical records and in-person evaluation by a dermatologist.

Methods

This investigation consisted of two components - (A) a cross-sectional study with a retrospective review of the medical records of all patients with a positive reverse-transcriptase polymerase chain reaction (RT-PCR) test for SARS-CoV-2 treated at Santa Casa de Misericórdia de Porto Alegre between March 2020 and November 2020, and (B) a prospective case series with in-person skin examination by an attending dermatologist of all patients admitted to COVID-19 wards between April 2021 and July 2021. The pattern of skin lesions and other variables were assessed.

Results

Information from 2968 individuals with COVID-19 was collected (2826 from the medical records and 142 from the in-person examination by a dermatologist). Of these, a total of 51 patients (1.71%) had COVID-19-related cutaneous lesions - 36 from the medical records group (1.27% of cutaneous manifestations) and 15 from the examinated group (10.56% of cutaneous manifestations). Of 51 patients, 15 (29.41%) died. There was no association between mortality and patterns of cutaneous manifestations. The variables male sex (p=0.021), intensive care unit (ICU) admission (p=0.001), and use of three or more antibiotics (p=0.041) were associated with higher mortality.

Conclusions

The risk factors, proven by our study, for mortality in patients with COVID-19 and cutaneous manifestations were male sex, ICU stays, and use of three or more antibiotics. Using the review of medical records as a tool for evaluating cutaneous manifestations related to COVID-19, there are about 10 times fewer occurrences when compared to in-person evaluation by a dermatologist.

## Introduction

Cutaneous findings in SARS-CoV-2 infection are polymorphic and may include rashes as well as urticarial, livedoid, purpuric, and pernio-like lesions [1-8]. Although some studies have reported an association between coronavirus disease 2019 (COVID-19) mortality and cutaneous manifestations, especially of vascular origin, this relationship remains unclear [9,10].

Cutaneous involvement in COVID-19 was first reported in February 2020 in China by Guan et al., who found that two of 1099 patients with laboratory-confirmed COVID-19 had had a rash as a sign of infection [11]. Since then, multiple case reports and case series of COVID-19 with cutaneous involvement have been published, with widely variable manifestations [2,4,5,12-14].

The pathophysiology of COVID-19 is multifactorial, and the pathogenesis of COVID-19-related cutaneous manifestations has yet to be fully established. A hypercoagulable state, with coagulopathy and thrombotic events, is present in the most severe cases, in addition to a massive systemic cytokine storm promoting activation of monocytes, macrophages, and cytotoxic CD8^+^ T cells, which leads to clinical deterioration [15].

Several prognostic factors have been described for COVID-19 severity and mortality, such as male sex, advanced age, obesity, smoking, comorbid chronic diseases, and instability of vital signs (e.g., hypoxemia) [16]. Studies have produced conflicting results regarding the correlation between mucocutaneous lesions and disease severity. While the mere presence of a skin lesion does not appear to be an indicator of severity in patients with COVID-19, some patterns of cutaneous involvement, particularly of vascular origin, are reported to be somewhat associated with greater severity [1,3,9,17].

The aim of the present study was to ascertain whether the pattern of skin lesions, age, sex, ethnicity, long-term medication use, arterial oxygen saturation (SaO_2_) at the first examination, setting of care, and number of medications used to treat SARS-CoV-2 infection are associated with mortality in patients with a confirmed diagnosis of COVID-19 and cutaneous manifestations of the disease. In addition, to evaluate the occurrence of cutaneous manifestations in patients with a confirmed diagnosis of COVID-19 through a review of medical records and in-person evaluation by a dermatologist.

## Materials and methods

The present investigation consisted of two components - a retrospective cross-sectional study and a prospective case series. In the cross-sectional study, the medical records of all patients over 18 years of age, of both sexes, who tested positive for SARS-CoV-2 by reverse transcriptase-polymerase chain reaction (RT-PCR) and were treated at Santa Casa de Misericórdia de Porto Alegre, a tertiary care university hospital in southern Brazil, between March 17, 2020 and November 17, 2020, were retrospectively reviewed. Patients whose records contained any description of skin-related complications were included in the sample. The following data were collected: age, sex, ethnicity, comorbidities, long-term medications, SaO_2_ at the first examination, medications used to treat COVID-19, setting of care (outpatient, inpatient ward, or intensive care unit {ICU}), description of skin lesions, and outcome (hospital discharge or death).

In the case series, all PCR-positive patients admitted to a dedicated COVID-19 ward were examined in person by an attending dermatologist between April 12, 2021, and July 12, 2021. Patients found to have any skin-related manifestations during this assessment were included in the sample. The purpose of this second group was to determine whether there would be a difference in the prevalence of cutaneous manifestations between assessments performed by non-dermatologists and by dermatologists. Both studies were approved by the Research Ethics Committees of the Universidade Federal de Ciências da Saúde de Porto Alegre (CAAE number 32217720.4.0000.5345, opinion 4,108,150) and Santa Casa de Misericórdia de Porto Alegre (CAAE number 32217720.4.3002.5335, opinion 4,237,991).

Dermatological findings related to COVID-19 were divided into three main patterns - vascular (pernio-like, livedoid, purpuric, and vasculitis-like), inflammatory (urticaria and exanthemas), and drug eruptions (attributable to drugs used in the management of COVID-19).

For statistical analysis, the variables were expressed as absolute and relative frequencies. Pearson’s chi-square test or Fisher’s exact test were used to test potential associations as appropriate. For polytomous variables, statistical significance was estimated on Fisher’s exact test by Monte Carlo simulation. In the presence of statistical significance, adjusted residual analysis was used to locate the significant associations. The level of significance was set at 5%, and all analyses were performed in SPSS version 21.0 (Armonk, NY: IBM Corp.).

## Results

**Figure 1:**
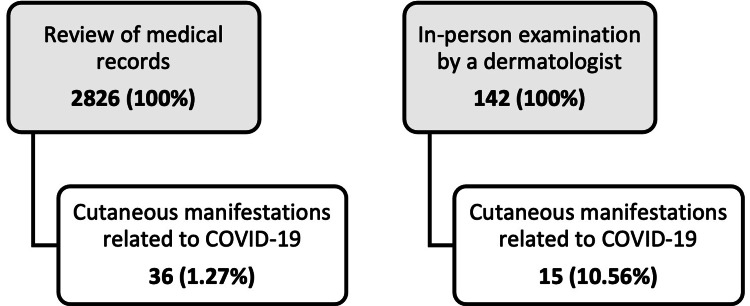
Comparison between the dermatological findings obtained through a review of medical records and those found in face-to-face hospital visits (p<0.001). COVID-19: coronavirus disease 2019

In the cross-sectional study, 2826 medical records were reviewed, and 36 patients (1.27%) had a report of COVID-19-related cutaneous manifestations. In the case series, 142 patients were examined in person by a dermatologist, and 15 (10.56%) had COVID-19-related cutaneous manifestations. There was a statistically significant difference (p<0.001) between the number of dermatoses found between the two groups (review of medical records and in-person examination by a dermatologist) (Figure 1).

**Figure 2:**
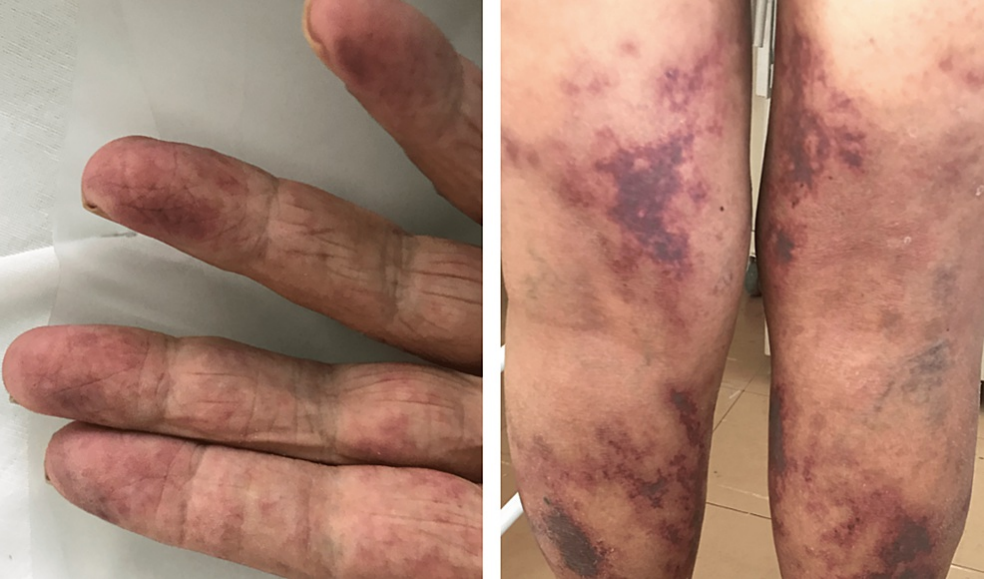
Vascular pattern: perniosis-like acrocyanosis (left) and vasculitis (right).

**Figure 3:**
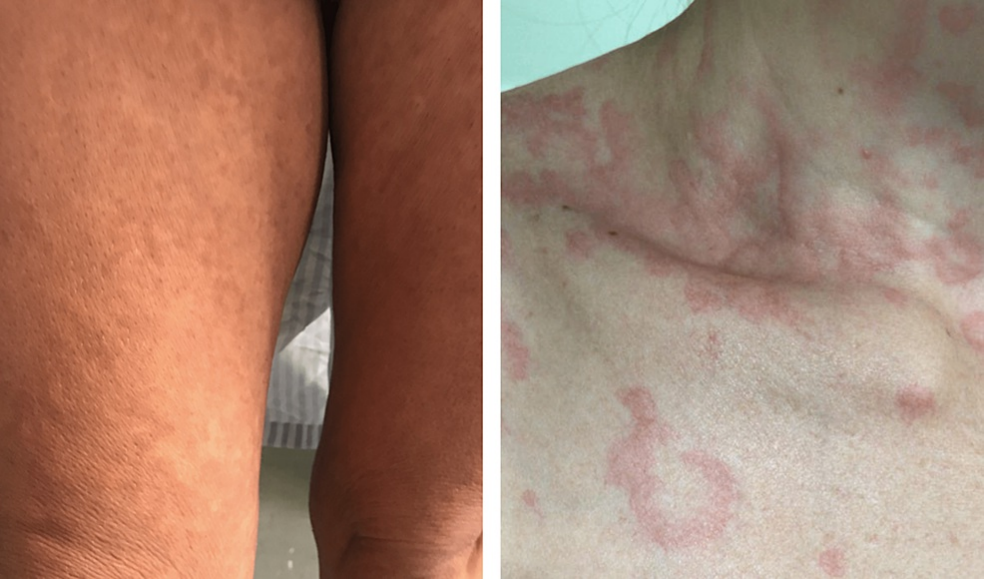
Inflammatory pattern: maculopapular exanthema (left) and urticarial eruption (right).

**Figure 4:**
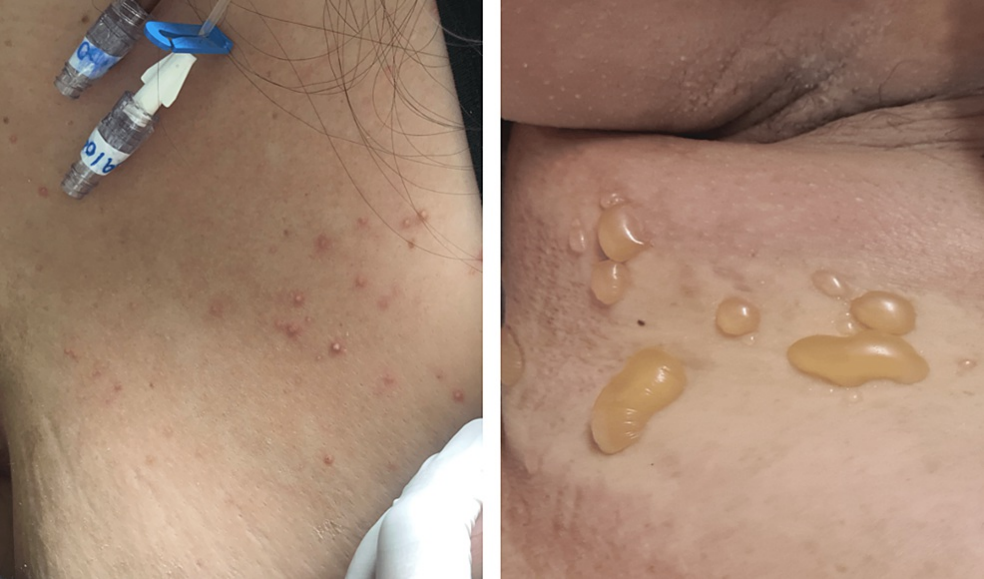
Drug eruption: acneiform eruption (left) and bullous SDRIFE (right). SDRIFE: symmetrical drug-related intertriginous and flexural exanthema

**Figure 5:**
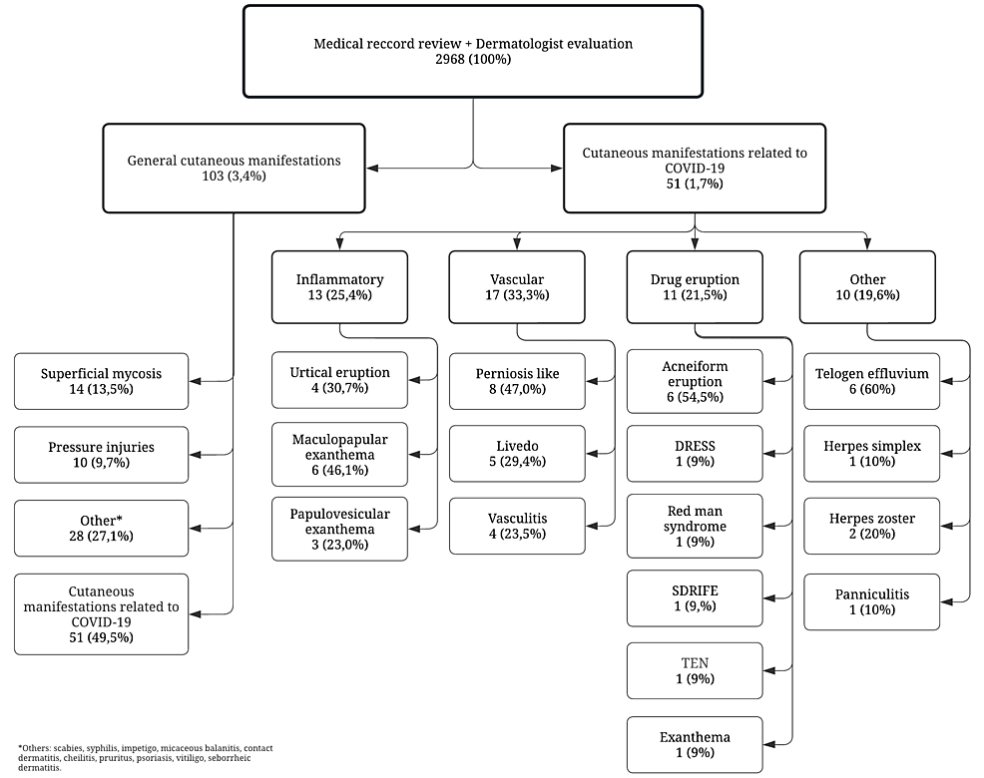
General cutaneous manifestations and cutaneous manifestations related to COVID-19 found in the medical record review and in the in-person evaluations by a dermatologist. COVID-19: coronavirus disease 2019; DRESS: drug reaction with eosinophilia and systemic symptoms; SDRIFE: symmetrical drug-related intertriginous and flexural exanthema; TEN: toxic epidermal necrolysis

Dermatological findings were mostly classified as having a vascular pattern (perniosis, livedo, vasculitis) (Figure 2), followed by inflammatory lesions (urticarial eruption, maculopapular exanthema, papulovesicular exanthema) (Figure 3) and drug eruptions (acneiform eruption, drug reaction with eosinophilia and systemic symptoms {DRESS}, RED man syndrome, symmetrical drug-related intertriginous and flexural exanthema {SDRIFE}, toxic epidermal necrolysis {TEN}, and exanthema) (Figure 4). Other dermatological manifestations, not classified in the three major patterns, but which were related to the context of COVID-19 were as follows: telogen effluvium, cutaneous lesions due to herpes zoster and herpes simplex, and panniculitis (Figure 5).

Among the dermatoses not directly related to COVID-19, the most registered were superficial fungal infections (tinea pedis, intertrigo due to fungal infection, mucocutaneous candidiasis), followed by wound and pressure ulcers (located in the sacrum, gluteus, calcaneus, ankle, and chin) (Figure 5).

Patients from both studies who had cutaneous manifestations were analyzed together to search for risk factors for mortality (n=51) (Table 1). There was no association of age, ethnicity, long-term medication use, or SaO_2_ at the first examination with mortality. Also, mortality did not differ significantly between the distinct patterns of cutaneous manifestations. Significantly more men died than women (73.3% vs. 26.7%, p=0.021). Use of three or more antibiotics (p=0.041) and need for ICU admission (p=0.001) were positively associated with death in these patients (Table 1).

**Table 1:** Mortality in patients with COVID-19 and cutaneous manifestations. COVID-19: coronavirus disease 2019 *Statistically significant association by adjusted residual analysis at the 5% significance level. **Missing data for some patients. ***Fisher’s exact test. ****Cutaneous findings that could not be categorized into any of the three patterns and were not included in this table: telogen effluvium (n=6), herpes simplex (n=2), herpes zoster (n=2), panniculitis (n=1).

Variables		Overall sample (n=51)	Deceased (n=15)	p-Value
Age	18-56 years	26 (51%)	7 (46.7%)	1.000
	>56 years	25 (49%)	8 (53.3%)	
Sex	Female	28 (54.9%)	4 (26.7%)	0.021
	Male	23 (45.1%)	11 (73.3%)*	
Ethnicity^**^	White	40 (85.1%)	12 (80%)	0.664***
	Non-white	7 (14.9%)	3 (20%)	
Chronic medications^**^	0-1 medication	21 (42.9%)	3 (21.4%)	0.110
	>1 medications	28 (57.1%)	11 (78.6%)	
SaO_2_ on arrival^**^	95 or high	32 (69.6%)	10 (66.7%)	1.000***
	<95%	14 (30.4%)	5 (33.3%)	
COVID-19 drugs	Symptomatic only	8 (15.7%)	0 (0%)	0.041***
	0-2 antibiotics	18 (35.3%)	4 (26.7%)	
	>2 antibiotics	25 (49.0%)	11 (73.3%)*	
Management setting	ICU	23 (45.0%)	12 (80%)*	0.001***
	Ward/floor	14 (27.5%)	3 (20%)	
	Home	14 (27.5%)	0 (0%)	
Cutaneous involvement pattern****	Inflammatory	13 (31.7%)	2 (15.4%)	0.066***
	Vascular	17 (41.5%)	9 (69.2%)	
	Drug eruption	11 (26.8%)	2 (15.4%)	

## Discussion

Several studies have associated clinical severity with the pattern of cutaneous lesions in COVID-19, with vascular skin manifestations being more commonly observed in patients with a more severe clinical course [1,3,9,10]. However, this association was not found in our overall sample (p=0.066). Perna et al. reported that vasculitis or thrombosis was suspected in almost 70% of patients with skin lesions, suggesting an association between cutaneous manifestations and systemic vascular damage in COVID-19 [18]. Skin biopsies showing perivascular infiltrates and microthrombi further support the correlation with disease severity [19].

Mortality was higher in men than in women. It has long been known that mortality rates are higher in men across all age groups and for most causes of death [20]. This has also been true for COVID-19, with reports that men are more likely to develop serious complications than women and progress to a fatal outcome [16,21-23]. A recent systematic review of the prognostic factors for severity and mortality in patients infected with SARS-CoV-2, including more than 200 studies, confirmed male sex as a risk factor [16].

Mortality was not associated with long-term use of two or more medications (p=0.110). Although there is evidence to suggest that certain comorbidities are risk factors for more severe disease and death, we found no information on increased risk specifically related to the number of long-term medications [16,22,24].

Hypoxemia, a marker strongly associated with in-hospital mortality, was not associated with mortality in our study [25]. This may have occurred because SaO_2_ was only recorded after the initial patient examination and admission to the hospital, when patients were clinically compensated, and because mortality was evaluated in a small subgroup of patients who, in addition to hypoxemia, had skin lesions.

Both ICU admission and the use of three or more antibiotics are generic criteria for disease severity, and, as expected, these two variables were associated with mortality in our study. As confirmed by Grasselli et al., mortality is higher in critically ill patients with COVID-19 who stay in the ICU [22].

There was a substantial difference between the number of dermatological findings obtained through the review of medical records and those found in the in-person examination by a dermatologist (1.27% vs. 10.56%, p<0.001), indicating that medical record review is not a suitable method to search for skin manifestations in patients infected with SARS-CoV-2, and specialist assessment is necessary to obtain more reliable data. Available systematic reviews show an overall frequency of 5.69% and 5.95% [3,12]. The prevalence found in our study exceeds these values when we only used in-person assessments performed by a dermatologist (10%), demonstrating that the number of cutaneous manifestations related to the virus is probably higher, but there is a lack of diagnoses in the absence of the specialist doctor. Despite this difference in the number of dermatoses, both the retrospective and prospective studies had very similar designs, with the same variables collected, thus allowing data aggregation, and providing a larger sample.

In summary, as the main clinical implications, the present study demonstrates the following: (1) COVID-19 can be directly or indirectly associated with the emergence of various dermatoses and a care team must be composed of a dermatologist to increase diagnostic capacity and maximize the therapeutic management of patients, and (2) in patients with dermatoses associated with COVID-19, the fact that they use three or more antibiotics and have the need for ICU management, increase the risk for the outcome of death.

Limitations

Limitations of our study remain the research conducted in a single university hospital center (southern Brazil). The small number of dermatoses found, even in a large sample, did not allow differentiation between skin patterns and prediction of clinical outcomes in patients with COVID-19.

## Conclusions

A comprehensive, retrospective, in-hospital observation of dermatological manifestations in patients with COVID-19 was performed, and one arm of the study had a prospective case series. There was a considerable difference in the frequency of dermatoses between the main study (review of medical records) and the aforementioned arm (in-person examination by an attending dermatologist). Additionally, there was no specific pattern of cutaneous manifestations found in our COVID-19-positive patients. Both constatations of our study demonstrate the importance of specialized assessment for the diagnosis of dermatoses in inpatients with SARS-CoV-2 infection.

The risk factors, proven by our study, for mortality in patients with COVID-19 and cutaneous manifestations were male sex, ICU stays, and use of three or more antibiotics. There were no differences between patterns of skin lesions regarding the risk of death. Inclusion of the assessment of cutaneous manifestations did not add value in terms of predicting the risk of death when using information obtained mainly from medical records, despite the large sample evaluated.
